# Context-Dependent Neural Modulations in the Perception of Duration

**DOI:** 10.3389/fnint.2016.00012

**Published:** 2016-03-08

**Authors:** Yuki Murai, Yuko Yotsumoto

**Affiliations:** ^1^Department of Life Sciences, The University of TokyoTokyo, Japan; ^2^Japan Society for the Promotion of ScienceTokyo, Japan

**Keywords:** time perception, fMRI, sub-second, supra-second, time reproduction, temporal context

## Abstract

Recent neuroimaging studies have revealed that distinct brain networks are recruited in the perception of sub- and supra-second timescales, whereas psychophysical studies have suggested that there are common or continuous mechanisms for perceiving these two durations. The present study aimed to elucidate the neural implementation of such continuity by examining the neural correlates of peri-second timing. We measured neural activity during a duration reproduction task using functional magnetic resonance imaging. Our results replicate the findings of previous studies in showing that separate neural networks are recruited for sub-versus supra-second time perception: motor systems including the motor cortex and the supplementary motor area for sub-second perception, and the frontal, parietal, and auditory cortical areas for supra-second perception. We further found that the peri-second perception activated both the sub- and supra-second networks, and that the timing system that processed duration perception in previous trials was more involved in subsequent peri-second processing. These results indicate that the sub- and supra-second timing systems overlap at around 1 s, and cooperate to optimally encode duration based on the hysteresis of previous trials.

## Introduction

Temporal information in different timescales is related to different behavioral functions, and processed by different neural systems (Buhusi and Meck, 2005). Previous studies have shown that there are two timing systems divided by a boundary of around 1 s (Lewis and Miall, 2006). Durations in the hundreds of milliseconds (i.e., sub-second) are involved in motor control (Merchant and Georgopoulos, 2006) and speech generation (Schirmer, 2004), whereas durations of a few seconds (i.e., supra-second) are critical for foraging (Kacelnik and Brunner, 2002) and decision-making (Sohn and Carlson, 2003). Previous psychological studies have reported that the coefficient of variation or the Weber fraction of the perceived duration changes at the boundary at around 1 s (Gibbon et al., 1997; Grondin, 2014), and that cognitive load affect differently for the sub- and supra-second timing performance (Rammsayer and Lima, 1991). Neuroimaging studies have revealed that sub- and supra-second timing also differ in terms of their neural implementations (Lewis and Miall, 2003b; Wiener et al., 2010). The processing network for sub-second durations mainly involves the motor system, including the supplementary motor area (SMA), the primary motor area, and the primary somatosensory area, whereas the supra-second network includes the prefrontal cortex, the posterior parietal cortex, and the basal ganglia, which are areas involved in attention and/or working memory (Lewis and Miall, 2006). In physiological studies, duration-tuned neurons have been reported for sub-second timing in such areas as the SMA-preSMA (Merchant et al., 2013a,b; Crowe et al., 2014) and the putamen (Bartolo et al., 2014), and for supra-second timing in the prefrontal cortex (Yumoto et al., 2011).

While previous neuroimaging studies have emphasized the distinctions between the sub- and supra-second timing systems (Pouthas et al., 2005; Jahanshahi et al., 2006; Lewis and Miall, 2006; Wiener et al., 2010; Hayashi et al., 2014), the nature of continuity between these two systems remains an open question. Psychologically, we can execute timing tasks whether the target duration is sub-second, supra-second, or around 1 s (i.e., peri-second). Seamless timing across different timescales cannot be realized without some intermediate or transitional state between the sub-second system and the supra-second system. This raises the question: how are peri-second durations processed in our brain? A few studies have indirectly dealt with this issue. A meta-analysis study reported the presence of brain regions that are activated in both sub- and supra-second timing (Wiener et al., 2010). Several functional magnetic resonance imaging (fMRI) studies have reported that BOLD activities in various brain regions correlate with event durations at timespans ranging from the milliseconds to seconds range (Morillon et al., 2009; Wencil et al., 2010; Coull et al., 2015). These fMRI studies, however, assumed a monotonic change of BOLD activity across sub- and supra-second timing, and did not directly investigate the distinction between sub- and supra-second timing. These studies suggest that continuities exist between the sub- and supra-second timing systems. However, no neuroimaging study has directly examined how these two distinct neural timing systems operate for peri-second durations.

One plausible solution for peri-second processing is that both the sub-second and supra-second systems are involved in processing peri-second durations, and therefore, peri-second durations are perceived through cooperation of these two systems. In such a framework, it is predicted that peri-second timing activated both the sub- and supra-second timing systems.

While the transition between timing systems might depend on the duration to be timed, the transition might also depend on the hysteresis of previous trials. It is well known that stimulus history induces biases in various timing tasks (Miyazaki et al., 2006; Jazayeri and Shadlen, 2010; Shi et al., 2013a). One example of these phenomena is the central tendency in timing, which can be described as follows: when various durations are presented in an intermixed order, relatively shorter durations are overestimated and relatively longer durations are underestimated. This phenomenon occurs across different timescales (Lejeune and Wearden, 2009): when various durations in the milliseconds-to-seconds range are intermixedly presented, shorter sub-second durations are overestimated, and longer durations in the supra-second range are underestimated. These phenomena suggest that duration perception depends on the history of recently presented durations.

Based on these psychophysical observations, which suggest the presence of a hysteresis-based timing mechanism, we hypothesized that peri-second processing relies on the timing system that predominately involves the processing system corresponding to the durations in previous trials. In other words, when peri-second trials are placed in between sub-second trials, the peri-second duration is encoded mainly by the sub-second system. On the other hand, when peri-second trials are placed in between supra-second trials, the peri-second duration is encoded mainly by the supra-second system.

Thus far, we have two independent hypotheses regarding peri-second processing. First, we hypothesized that both the sub- and supra-second timing systems are recruited for peri-second timing. Second, we hypothesized that the durations in previous trials affects peri-second processing. To test these hypotheses, we measured neural activity while subjects performed a duration reproduction task using fMRI.

## Materials and Methods

### Subjects

Twenty-one healthy volunteers (12 males and 9 females, 18–23 years old) participated in the fMRI experiment. All participants gave written informed consent for their participation in the experimental protocol, which was approved by the institutional review boards of The University of Tokyo. All subjects reported to have normal or corrected-to-normal vision.

### Procedure

**FIGURE 1:**
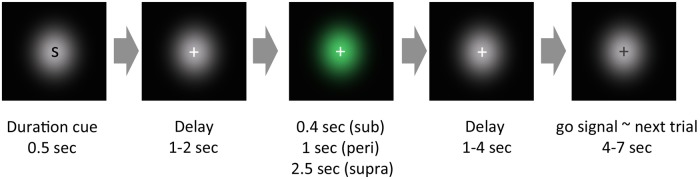
**Schematic of an experimental trial.** In our experimental paradigm, each trial began with a cue presentation, which roughly informed the subjects of the cue duration to be presented. After a pseudorandom delay, a green Gaussian blob was presented for a certain duration. After another pseudorandom delay, the color of the fixation cross changed from white to black. After this change of color, subjects reproduced the perceived duration of the green stimulus by pressing a button.

All visual stimuli were generated using MATLAB with the Psychophysics Toolbox (Brainard, 1997). A schematic of a trial is shown in **Figure 1**. The task was to reproduce the duration of the visually presented Gaussian luminance blob. In the experiment, each trial began with a cue presentation, which informed the subject of the duration to be presented: a single character, S, M, or L, was presented for the sub-, peri-, or supra-second condition, respectively. After a pseudorandom delay (1–2 s), a green Gaussian blob was presented for a certain duration. The stimulus duration was 0.4 s for the sub-second condition, 1 s for the peri-second condition, or 2.5 s for the supra-second condition. Subjects were not explicitly informed that stimulus durations within each condition were constant. After a pseudorandom delay (1–4 s), the color of the fixation cross changed from white to black (go signal). After viewing this go signal, subjects reproduced the perceived duration of the green stimulus by making a sustained button press that lasted for the perceived duration. All subjects used their left thumb to press the response button.

Subjects completed 10 fMRI runs in total. In half of the runs, the peri-second trials were intermixed with the sub-second trials, and in the other half of the scans, the peri-second trials were intermixed with the supra-second trials. Sub- and peri-second runs contained 20 trials for each duration, resulting in the subjects completing 100 trials in total for each duration within five runs. Supra- and peri-second runs contained 16 trials for each duration, resulting in the subjects completing 80 trials in total within the other five runs. The runs with sub- and peri-second trials and the runs with supra- and peri-second trials were performed alternately, and the type of run that began each experiment was counterbalanced across subjects. In subsequent contrast analysis, one randomly chosen sub- and peri-second run was discarded to equalize the number of trials used for contrast calculation for all conditions. Therefore, 80 trials for each condition were used in the analysis.

We added a duration cue at the beginning of a trial for the following reason: each run included two distinct durations, and therefore, without the duration cue, subjects could judge whether the stimulus duration was the longer one or the shorter one during stimulus presentation. For example, if no duration cue was presented in the supra-second trials which were intermixed with the peri-second trials, subjects could judge that the presented stimulus duration was the longer one, as it lasted some duration beyond 1 s. By adding the duration cue, which informed the subjects in advance of the duration to be presented, we aimed to prevent the possibility that the brain activity induced by categorical judgment about the stimulus duration would contaminate timing-induced brain activity during stimulus presentation.

### MRI Data Acquisition

MRI data was acquired using a 3T MRI system (Magnetom Prisma, Siemens, Erlangen, Germany), equipped with a 64-channel head coil. For each subject, a high-resolution anatomical scan (MPRAGE) was performed. The total data acquisition time for the anatomical scan was 4.7 min (*TR* = 2 s, *TE* = 2.9 ms, flip angle = 9 °, matrix size = 240 × 256 × 176, spatial resolution = 1 mm × 1 mm × 1 mm). EPI sequences (*TR* = 2 s, *TE* = 30 ms, flip angle = 90°) were used to obtain functional MR images. Thirty-nine contiguous slices (3 mm × 3 mm × 3.5 mm, with 10% gap) oriented parallel to the AC-PC plane were acquired to cover the whole brain, using an interleaved slice acquisition sequence. The total time for each functional run was 6.2 min.

### Data Analysis

Data were analyzed with FS-FAST and FreeSurfer (http://surfer.nmr.mgh.harvard.edu) software. For image preprocessing, all functional images were head motion corrected, slice-time corrected, spatially smoothed with a Gaussian kernel of 8.0 mm (FWHM). The mean intensity for the entire functional volume was computed for each scan. The global mean of the entire brain was rescaled so that the same mean was set across scans. All functional data were registered to the individual anatomically reconstructed brain.

Hemodynamic responses evoked by different task components were modeled as single events convolved with a canonical hemodynamic response function. Events time-locked to the presentation of the stimuli and the onset time of reproduction were defined separately for each duration condition, that is, sub-second, peri-second intermixed with sub-second, peri-second intermixed with supra-second, and supra-second durations. This resulted in eight distinct event types: 2 task components (stimulus/reproduction) × 4 durations. Duration of the stimulus event was defined as duration of the stimulus presentation, and duration of the reproduction event was defined as the reproduced duration. In the present study, we used a rapid event-related design. In the experiment, events were closely spaced, resulting in substantially overlapped hemodynamic responses. However, previous studies have shown that the underlying hemodynamic responses can be computationally deconvolved by randomly jittering inter-event intervals and under the assumption of linearity (Dale and Buckner, 1997; Burock et al., 1998). Using relatively long and varying intervals between the stimulus interval and the reproduction interval allowed temporal deconvolution of the BOLD response for the stimulus interval and the reproduction interval (Coull et al., 2008). To examine brain activity manifest by duration encoding, events in the stimulus phase were used for the following contrast analysis. We eliminated activation elicited by duration reproduction from the contrast analysis, because any change of BOLD response depending on duration condition can be attributable to the effect of the variation of reproduced durations in each duration condition, or memory decay derived from the variation of time from trial onset.

In the analysis, we first identified brain regions related to sub- and supra-second timing by contrasting the BOLD response for the sub-second stimulus presentation and that for the supra-second stimulus presentation. Then, to identify regions involved in peri-second timing, we compared the BOLD response for the peri-second stimulus presentation to either the BOLD response for the sub- or the supra-second stimulus presentation. In the experiment, the sub- and supra-second trials appeared in half of the total of 10 runs, while the peri-second trials appeared in all runs. To compensate for the number of trials needed to calculate the BOLD contrast, we compared the BOLD responses between the trials presented in the same type of runs: the BOLD responses for the sub-second stimulus were contrasted to that for the peri-second stimulus intermixed with the sub-second trials, while the BOLD response for the supra-second stimulus were contrasted to that for the peri-second stimulus intermixed with the supra-second trials. Finally, to examine the hysteresis of the previous trials, the BOLD response for the peri-second stimulus intermixed with the sub-second trials was compared to the BOLD response for the peri-second stimulus intermixed with the supra-second trials. These two types of peri-second conditions had the same stimulus and same duration, and were different in terms of intermixed trials that were either sub- or supra-second trials.

One of the difficulties in neuroimaging studies of time perception is the use of control tasks. Some studies have compared brain activity of subjects while they were executing a timing task with that of subjects executing a control task (e.g., color task) in response to the same stimuli (Coull et al., 2004; Morillon et al., 2009). These studies defined the regions that exhibited greater activities in the timing task as “timing-related” regions. However, Livesey et al. (2007) reported that some of these “timing-related” activities are attributable to differences in the difficulties of the timing versus control tasks (Livesey et al., 2007). To eliminate the effect of task difficulty in the control task in the present study, we did not employ any explicit control tasks. Alternatively, we directly compared brain activities when subjects were timing the presented stimulus. Given that the stimuli used in all conditions were the same Gaussian luminance blobs and the task was always to reproduce the presented duration, the only thing that was different across conditions was the stimulus duration.

## Results

### Behavioral Data

The mean and SD of the reproduced duration was 657.0 ± 166.6 ms for the sub-second trials, 1223.8 ± 215.4 ms for the peri-second trials intermixed with the sub-second trials, 1348.0 ± 265.4 ms for the peri-second trials intermixed with the supra-second trials, and 2358.1 ± 289.7 ms for the supra-second trials.

To test the contextual effect of previous trials, we first conducted a two-way repeated-measures ANOVA with run type (sub- and peri-second run or peri- and supra-second run) and relative duration within a run (short or long) as factors. To compensate the difference of stimulus durations across conditions, the ratio of the reproduced duration to the stimulus duration was tested as an index of duration estimation error. If the central tendency occurs, the relatively short duration within a run should be overestimated, and the relatively long duration within a run should be underestimated. That is exactly what we found.

**FIGURE 2:**
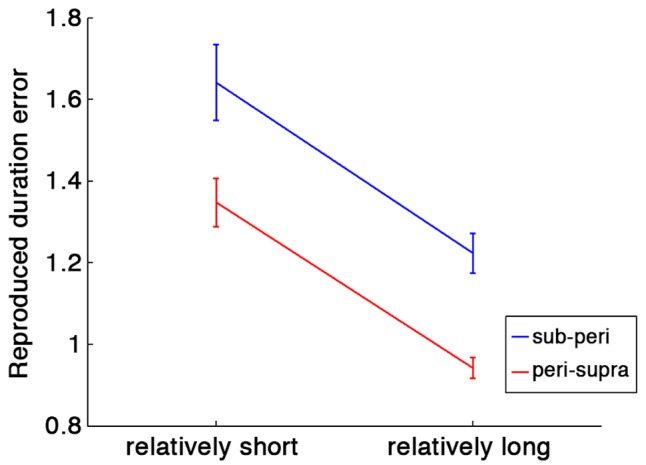
**Duration estimation errors calculated as ratios of the reproduced duration to the stimulus duration.** Blue line indicates the duration estimation errors in runs with sub- and peri-second trials, and red line indicates the duration estimation errors in runs with peri- and supra-second trials. Error bars indicate standard errors (SE). Horizontal axis represents relative duration within each run: in runs with peri- and supra-second trials, for example, the peri-second durations are the relatively shorter durations and the supra-second durations are the relatively longer durations.

As shown in **Figure 2**, there were a significant main effect of relative duration within a run [*F*(1,80) = 46.84, *p* < 0.001] and of run type [*F*(1,80) = 22.84, *p* < 0.001]. No significant interaction was found [*F*(1,80) = 0.01, *p* = 0.91]. These results indicate that the central tendency occurred in both run types, and that durations were more overestimated in runs with sub- and peri-second trials than in runs with supra- and peri-second trials.

Furthermore, we tested the contextual effect in peri-second timing. The reproduced duration for the peri-second trials intermixed with the supra-second trials was significantly longer than those for the peri-second trials intermixed with the sub-second trials [*t*(20) = 4.72, *p* < 0.0001, *d* = 1.03]. This tendency was highly consistent across individuals (19 out of 21 subjects exhibited this tendency). This result indicates that the duration of previous trials affected the perceived duration of the peri-second stimulus. It should be noted that the reproduced durations for peri-second trials were longer than 1 s both for the peri-second trials intermixed with the sub-second trials [*t*(20) = 4.76, *p* = 0.0001, *d* = 1.04] and for the peri-second trials intermixed with the supra-second trials [*t*(20) = 6.01, *p* < 0.0001, *d* = 1.31]. The overestimation in reproduced duration for sub- to peri-second durations is consistent with a previous study (Shi et al., 2013b).

### fMRI Data

**FIGURE 3:**
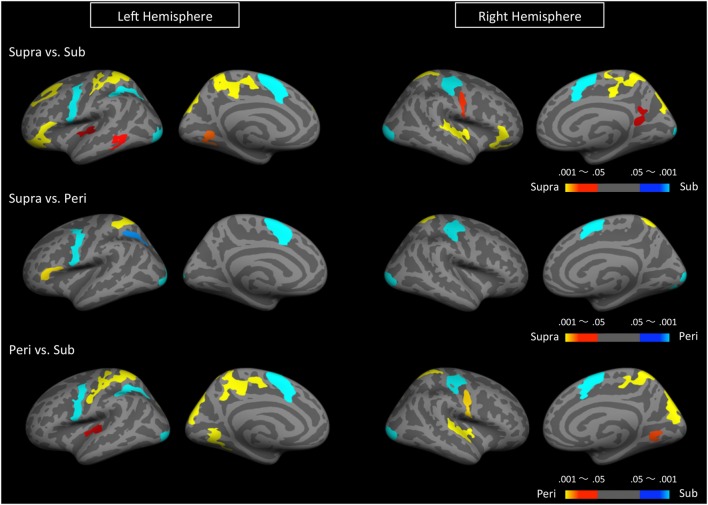
**Significantly activated clusters in sub-, peri-, and supra-second timing.** The color scales indicate the cluster-wise corrected *P*-values.

We first determined brain regions that exhibited different activation patterns between the sub- and the supra-second timing. When the subjects timed sub-second stimuli, the bilateral SMA, the bilateral visual cortex, the left premotor area, the right intra-parietal sulcus (IPS), and the right precentral and postcentral region were activated. By contrast, when subjects timed supra-second stimuli, significant activations in the inferior frontal gyrus (IFG), the superior frontal gyrus (SFG), the superior parietal cortex, the superior temporal cortex, the lingual gyrus, the putamen, and the ventral cerebellum were observed (**Figure 3**, **Table 1**). The timing networks we described are largely consistent with previously reported sub- and supra-second timing networks (Lewis and Miall, 2003b; Wiener et al., 2010) and confirm that sub-second timing versus supra-second timing depend on distinct brain networks. However, it should be noted that we observed the activation of the cerebellum in supra-second timing, whereas the cerebellum is often reported to be involved in the sub-second timing network (Lewis and Miall, 2006; Wiener et al., 2010).

**Table 1:** Brain regions significantly activated by either sub-, peri-, or supra-second timing.

Anatomical description	Side	MNI coordinates				MNI coordinates				MNI coordinates			
		*X*	*Y*	*Z*	*P*-value	*X*	*Y*	*Z*	*P*-value	*X*	*Y*	*Z*	*P*-value
		Supra > Sub				Supra > Peri				Peri > Sub			
**Frontal**													
Superior frontal gyrus	L	−22	22	49	0.0001								
Inferior frontal gyrus	L	−34	45	−11	0.0001	−51	32	4	0.0013				
	R	51	33	−5	0.0001								
Precentral	R	43	−10	37	0.0086								
**Parietal**													
Paracentral	L	−10	−17	48	0.0001					−10	−17	48	0.032
Postcentral	R									63	−8	12	0.025
Superior parietal cortex	L	−18	−86	37	0.0001	−31	−46	56	0.0001	−18	−86	37	0.0001
	R	22	−82	41	0.0001	22	−51	60	0.0002	21	−83	41	0.0001
Precuneus	R	15	−46	56	0.0001					12	−47	65	0.0001
	R	10	−54	11	0.027								
**Temporal**													
Superior temporal cortex	L	−44	−23	3	0.039					−53	−19	4	0.034
	R	38	−13	4	0.0001					54	−24	6	0.0001
Middle temporal cortex	L	−60	−52	−3	0.013								
**Occipital**													
Lingual	L	−15	−61	0	0.005					−14	−61	0	0.0001
	R									8	−63	5	0.0068
**Subcortical**													
Cerebellum	L	−28	−41	−47	0.0001	−37	−47	−30	0.0001	−30	−43	−47	0.0001
	R	18	−55	−55	0.0016					20	−51	−57	0.0026
Putamen	R	22	1	−8	0.0158								
		
		**Sub > Supra**				**Peri > Supra**				**Sub > Peri**			
**Frontal**													
Pre-SMA/SMA	L	−11	11	43	0.0001	−6	0	64	0.0001	−7	−2	64	0.0001
	R	7	3	66	0.0001	8	1	57	0.0007	8	3	66	0.0001
Precentral	L	−58	6	27	0.0001	−53	−4	44	0.0001	−57	6	27	0.0001
	R	37	−19	57	0.0001	37	−20	59	0.0001	37	−19	57	0.0001
**Parietal**													
Supramarginal gyrus	L	−41	−43	39	0.0001					−44	−45	40	0.0001
Superior parietal cortex	L					−27	−61	45	0.003				
**Occipital**													
Visual cortex	L	−26	−96	−8	0.0001	−26	−96	−8	0.0001	−27	−96	−8	0.0001
	R	28	−97	−5	0.0001	27	−98	−5	0.0001	24	−99	−8	0.0001

*MNI coordinates of peak activation and cluster-wise corrected P-values are presented for each cluster.*

Secondly, to determine the brain regions activated when subjects were timing the peri-second duration, we calculated the contrast between peri- and supra-second timing, and the contrast between peri- and sub-second timing (**Figure 3**, **Table 1**). In comparison with sub-second timing, peri-second timing activated the superior parietal cortex, the superior temporal cortex, the lingual gyrus, and the ventral cerebellum. These areas were mostly activated in supra-second timing, and were therefore included in the supra-second system. In contrast, when compared to supra-second timing, peri-second timing activated the SMA, the precentral area, the supramarginal gyrus, and the visual cortex. These areas were mostly activated in sub-second timing, and therefore included the sub-second system. As described above, peri-second timing thus activated both the sub- and the supra-second timing networks. These results suggest that duration at the boundary between the sub- and the supra-second is processed by a combination of the sub- and the supra-second systems.

**FIGURE 4:**
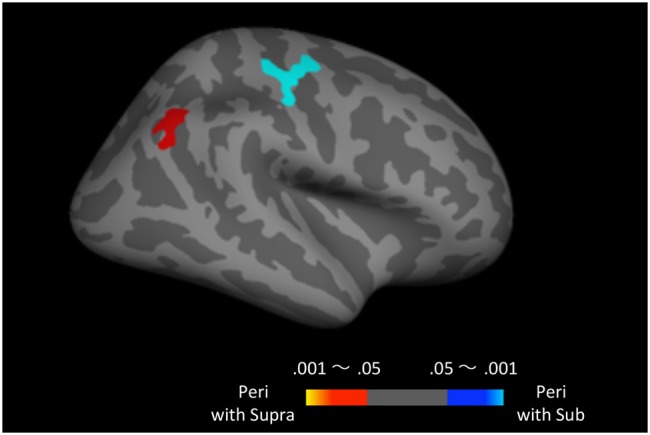
**Peri-second trials intermixed with supra-second trials versus peri-second trials intermixed with sub-second trials.** The color scales indicate the cluster-wise corrected P-value.

Finally, to examine the effect of recent trials on peri-second processing, we compared brain activity for peri-second timing intermixed with sub-second timing with brain activity for peri-second timing intermixed with supra-second timing (**Figure 4**, **Table 2**). The right precentral and postcentral areas were more activated in peri-second trials intermixed with sub-second trials. These areas were also activated in sub-second timing, and are thus thought to be included in the sub-second system. In contrast, the right inferior parietal lobule (IPL) was more activated for the peri-second trials intermixed with supra-second trials. The right IPL did not exhibit differential activity between sub- and supra-second timing in the present study, hence, there is no simple interpretation of the contextual effect in the right IPL. The right IPL has been linked to supra-second timing in previous studies (Lewis and Miall, 2002; Macar et al., 2002), therefore, we speculate that the right IPL activation in peri-second trials intermixed with supra-second trials might reflect the involvement of the supra-second timing system. These results suggest that the timing network which was activated by the recently presented durations predominates in peri-second timing. More specifically, when the subject frequently times sub-second durations, the peri-second duration is processed with a larger contribution from the sub-second system; when the subject frequently times supra-second durations, the peri-second duration is processed with a larger contribution from areas that have previously been associated with supra-second timing.

**Table 2:** Brain regions that exhibited context-dependent time processing.

		MNI coordinates			
Anatomical description	Side	*X*	*Y*	*Z*	*P*-value
		Peri with Supra > Peri with Sub			
Inferior parietal lobule	R	48	−60	41	0.0222
		Peri with Sub > Peri with Supra			
Precentral	R	38	−12	63	0.0001

*BOLD contrasts were calculated between peri-second trials intermixed with supra-second trials and peri-second trials intermixed with sub-second trials.MNI coordinates of peak activation and cluster-wise corrected P-values are presented for each cluster.*

## Discussion

In the present study, we examined the neural correlates of duration perception in the milliseconds-to-seconds time range using fMRI. Our results indicated that: (1) distinct brain networks are involved in sub- versus supra-second timing (in accordance with previous studies), (2) both the sub- and supra-second timing networks work in cooperation in encoding peri-second durations, and (3) the processing mechanism for peri-second durations is context dependent in that peri-second timing relies more on the timing system that processed durations presented in recent trials.

### Distinctions Between the Sub-second System and the Supra-second System

By comparing brain activities during sub-second timing and those during supra-second timing, we confirmed the presence of two separate timing systems for these two time ranges, as previous studies have reported (Lewis and Miall, 2003b; Pouthas et al., 2005; Jahanshahi et al., 2006; Wiener et al., 2010; Hayashi et al., 2014). Moreover, the activity patterns observed for sub- and supra-second timing in the present study were largely consistent with previous studies. Frontal and posterior parietal areas of the cortex, which are related to working memory or attention, were involved in supra-second timing, while motor and somatosensory systems, including the SMA, postcentral, and precentral areas, were activated in sub-second timing.

One exception might be our observation of activation in the cerebellum during supra-second timing. While several studies have reported that the cerebellum engages in sub-second timing (Lewis and Miall, 2006; Wiener et al., 2010), a meta-analysis by Lewis and Miall (2003b) reported that the cerebellum also contributes to supra-second timing. Thus, the role of the cerebellum in duration perception is still under debate. Harrington et al. (2004) showed that patients with cerebellar damage exhibited greater timing-related variability in a time reproduction task, but not in a time-perception task, and that this timing-related variability correlated with working memory performance. They concluded that the cerebellum might process task-relevant cognitive information when it involves the motor-output system. The observation of cerebellar activation in supra-second timing in the present study is consistent with their findings. Further study will be needed regarding the role of the cerebellum in timing perception in the milliseconds-to-seconds range.

### Transitional State in Peri-second Processing

In the present study, the peri-second duration preferentially activated the sub-second system versus the supra-second system, or vice-versa, depending on context. These results suggest that temporal processing for the peri-second duration relies on a transitional state, in which both the sub- and supra-second timing systems work in cooperation.

Behavioral results showed that the reproduced durations for both peri-second conditions were greater than 1 s. The overestimation of peri-second durations in the reproduction task is also reported by a previous study (Shi et al., 2013b). The over-reproduction and the simultaneous activation of the sub- and supra-second system in peri-second timing might raise a question regarding the fluctuation of the categorical boundary between the sub- and supra-second systems. The durations used in the present study were selected based on the assumption that the boundary between the sub- and supra-second timing systems lies at around 1 s (Lewis and Miall, 2006). In actuality, there exist controversies over the precise boundary between the two systems. A psychological meta-analysis study reported that the boundary lies at around 1500 ms (Gibbon et al., 1997), other researchers suggested that the boundary lies at around 500 ms (Rammsayer and Lima, 1991; Rammsayer, 1999). In the present study, we showed that both the sub- and supra-second systems are recruited for peri-second timing. If a boundary lies either at 1500 ms or 500 ms, and two timing systems distinctly switch their operation by this precise boundary, the simultaneous activation of the sub- and supra-second timing systems would not be observed at 1 s. The present results can be explained if we assume the boundary exists precisely at around 1 s, however, this assumption is not very congruent with previous psychological studies. Rather, these results imply another possibility that the durations that the sub- and supra-second timing systems can process widely overlap at around 1 s. Previous psychological and neuroimaging studies have reported that different timing system is recruited depending on the task and stimulus modalities (Lewis and Miall, 2003b; Merchant et al., 2008, 2013a; Wiener et al., 2010). The categorical boundary between the sub- and supra-second intervals might fluctuate within relatively widely overlapped durations between the sub- and supra-second timing systems, depending on the stimulus and task features. Because we tested only 1-s duration in the present study, we cannot dissociate whether two timing systems have a precise boundary or widely overlap around 1 s. Further study will be needed to examine the transition from the sub-second system to the supra-second system.

Many studies, including the present study, have compared neural correlates of sub- and supra-second timing by using distinctly different durations (Lewis and Miall, 2003a; Pouthas et al., 2005; Jahanshahi et al., 2006). In these cases, subjects were able to make a categorical judgment regarding whether the presented stimulus duration is relatively shorter or longer within an experimental session. Therefore, one might argue that the sub-second and supra-second systems do not code the absolute duration, but rather the relative duration within an experimental session. That is, the so-called “sub-second” system might process relatively shorter durations, while the so-called “supra-second” system might process relatively longer durations within a session. If this is indeed the case, it implies that the activation of the sub- and the supra-second systems in peri-second timing might not represent a transitional state between the sub- and supra-second systems.

If the “sub-second” system and the “supra-second” system, respectively, code “relatively shorter” and “relatively longer” durations, the peri-second trials intermixed with the sub-second trials would activate the supra-second system, because the peri-second trials had a relatively longer duration in runs with sub- and peri-second trials. Similarly, the peri-second trials intermixed with the supra-second trials would activate the sub-second system, because peri-second trials are relatively shorter in duration in runs with the supra- and the peri-second trials. However, our results contradicted these predictions, and therefore, relative duration coding by the sub- and supra-second systems is unlikely. It is certainly a possibility that the difference between the two peri-second conditions was not optimized to detect brain activity coding relative durations. However, in addition to the present results, previous studies have suggested that the sub- and supra-second systems code absolute durations: distinct activation in the sub- and supra-second systems has been reported even when multiple durations are not used in the task, and therefore, no information regarding relative duration was included in this task (Lewis and Miall, 2003a; Wiener et al., 2010). Thus, on the whole, it is unlikely that the sub- and supra-second system code relative duration information. Therefore, activation of both the sub- and supra-second systems in peri-second timing suggests that the peri-second duration perception system is a transitional state between the sub- and supra-second systems.

Such a transitional timing mechanism between different timescales might enable a seamless representation of time. Many psychological and neuroimaging studies have investigated the distinctions between the sub-second and supra-second systems using temporal tasks with various durations spanning different timescales (Gibbon et al., 1997; Rammsayer, 1999; Lewis and Miall, 2003a, 2009; Koch et al., 2009; Hayashi et al., 2014). These studies found there were differential brain activities or behavioral measures between sub- and supra-second timing, indicating that distinct psychological or neural mechanisms are recruited for sub- and supra-second timing. An oversight of these studies was that subjects executed the same timing tasks for durations of different timescales. The fact that the human observers can execute timing tasks whether the target duration is sub-second or supra-second suggests the presence of continuous or common mechanisms across the sub-second system and the supra-second system. Based on confirmatory factor analysis, Rammsayer and Troche (2014) proposed a hierarchical mechanism of time perception in which an interval-independent superordinate processing system controls the sub- and supra-second timing mechanisms. This superordinate common mechanism is certainly a possible means to realize seamless time perception across different timescales. However, the transitional processing mechanism in the peri-second duration suggested by the present study represents another possibility. Even if no superordinate system exists, the continuity of the sub-second system and the supra-second system in the peri-second range might enable the execution of timing tasks across different timescales. It should be noted that these two possibilities are not mutually exclusive, and further research is necessary to determine how these common or continuous mechanisms interact with each other.

### Context-Dependent Modulations of Peri-second Processing

Our results indicate that the processing mechanism used to perceive peri-second durations changes context-dependently; that is to say, peri-second timing relies more on the timing system which processed durations presented in recent trials. We also observed this contextual effect in the behavioral results. The reproduced duration for the peri-second trials was longer when they were intermixed with the supra-second trials, compared to when they were intermixed with the sub-second trials.

While peri-second timing intermixed with sub-second timing activated the right precentral area that was also activated during sub-second timing, peri-second timing intermixed with supra-second timing activated the right IPL that was not activated during supra-second timing. Hence, there is no simple interpretation of the contextual effect in the right IPL. Previous studies have reported activation in the right IPL during supra-second time reproduction tasks (Lewis and Miall, 2002; Macar et al., 2002). Moreover, the IPL has anatomical connections with the inferior frontal cortex and the auditory cortex (Caspers et al., 2011, 2013), which were activated in supra-second timing in the present study. Therefore, we speculate that the right IPL activation in peri-second timing intermixed with supra-second timing might reflect the involvement of the supra-second timing system.

In the present study, context-dependent encoding of time was observed only in the right hemisphere. It is known that various timing tasks activate predominantly right-sided networks (Rao et al., 2001; Battelli et al., 2007; Coull et al., 2013). In particular, the right posterior parietal lobe, where greater activation was found in peri-second trials intermixed with supra-second trials, has been proposed to be a core region for timing (for a review, Battelli et al., 2007). Regions surrounding the central sulcus correspond to the primary motor and primary somatosensory areas, where greater activation was found in peri-second trials intermixed with sub-second trials, have also been reported to engage in the sub-second timing (Lewis and Miall, 2003b). It should be noted that all the contrasts found in the present analysis were computed with regressors time-locked to the stimulus presentation. Therefore, precentral (primary motor area) and postcentral (primary somatosensory area) activation in the peri-second duration intermixed with the sub-second duration would not reflect differences in reproduced durations between two peri-second conditions.

Even though contrasting activation patterns were computed by regressors for the stimulus presentation phase, the difference in brain activity between two peri-second conditions corresponded to the reproduced duration. The peri-second trials intermixed with sub-second trials activated the precentral area, which is a part of the sub-second system observed in the present study, and the reproduced duration of these trials was shorter than the reproduced duration of the peri-second trials intermixed with supra-second trials, which activated the right IPL, which has been linked to supra-second timing in previous studies. Almost all of the previous neuroimaging studies examining neural correlates of perceptual duration used illusions of time, in which looming, moving, or flickering visual stimuli appeared to be longer than static stimuli (Wittmann et al., 2010; Bueti and Macaluso, 2011; Herbst et al., 2013, 2015). Because these studies manipulated the perceptual duration by changing stimulus features, whether the detected brain activities were related to changes in perceptual duration or stimulus features was not dissociable. In contrast, in the present study, differential brain activity that depended on the hysteresis of durations in previous trials was detected between physically identical visual stimuli. Therefore, the brain activity detected between two peri-second conditions can be attributed to context-dependent timing processing and the corresponding change of the perceptual duration.

Studies with Bayesian modeling have revealed that the temporal variability of perceived duration induces the contextual effect such as the central tendency (Jazayeri and Shadlen, 2010; Shi et al., 2013a; Petzschner et al., 2015). For the time reproduction task, when a single interval is presented and then subjects reproduce its duration, the temporal variability of the reproduced duration is larger compared to when multiple intervals are presented (Grondin, 2012, 2014). The contextual effect of the reproduced duration observed in the present study might reflect the inherent large temporal variability of the single interval reproduction task and Bayesian inference process. The context-dependent modulations of timing systems suggest that the brain optimally encodes stimulus duration based on the history of previous trials, as many psychophysical and modeling studies have suggested (Lejeune and Wearden, 2009; Jazayeri and Shadlen, 2010; Cicchini et al., 2012; Shi et al., 2013a; Petzschner et al., 2015). The present study thus found the neural correlate of these proposed context-dependent timing systems. These systems would help to efficiently encode duration under noisy conditions and in different temporal contexts, and might be responsible for Bayesian process in time perception.

Previous studies have shown that the timing system that is used depends not only on stimulus duration, but also on various stimulus features and/or task dimension. Whether or not the duration is defined by movement, whether a stimulus is continuous or not (Lewis and Miall, 2006), and possibly stimulus modality (Yuasa and Yotsumoto, 2015) can all modulate the selection of the timing network. In addition to these stimulus or task features, the hysteresis of previous trials is also a factor for encoding stimulus duration optimally.

## Conclusion

Using fMRI, we examined neural timing systems in milliseconds-to- seconds range, with a focus on the transition from the sub-second timing system to the supra-second timing system. We found that processing of the peri-second duration operates via a transitional state, in which both the sub- and supra-second timing systems work in cooperation. Furthermore, the processing mechanism of the peri-second duration changes context-dependently, relying more on the timing system that processed durations presented in recent trials.

## Author Contributions

YM and YY designed and performed the experiments. YM analyzed the data. YM and YY wrote the manuscript.

## Conflict of Interest Statement

The authors declare that the research was conducted in the absence of any commercial or financial relationships that could be construed as a potential conflict of interest.
